# Prognostic significance of p16 & p53 immunohistochemical expression in triple negative breast cancer

**DOI:** 10.1186/s12907-018-0077-0

**Published:** 2018-10-03

**Authors:** Atif Ali Hashmi, Samreen Naz, Shumaila Kanwal Hashmi, Zubaida Fida Hussain, Muhammad Irfan, Erum Yousuf Khan, Naveen Faridi, Amir Khan, Muhammad Muzzammil Edhi

**Affiliations:** 1 Liaquat National Hospital and Medical College, Karachi, Pakistan; 2 CMH Institute of Medical Sciences, Multan, Pakistan; 3 Kandahar University, North, Kandahar 3802 Afghanistan; 4 Brown University, Providence, RI USA

**Keywords:** p53, p16, Triple negative breast cancer

## Abstract

**Background:**

p16 and p53 genes are frequently mutated in triple negative breast cancer & prognostic value of these mutations have been shown; however, their role as immunohistochemical overexpression has not been fully validated. Therefore we aimed to evaluate the association of p16 and p53 overexpression in triple negative breast cancer with various prognostic parameters.

**Methods:**

Total 150 cases of triple negative breast cancers were selected from records of pathology department archives that underwent surgeries at Liaquat National hospital, Karachi from January 2008 till December 2013. ER, PR and Her2neu immunohistochemistry were re-performed to confirm triple negative status. p16 & p53 immunohistochemistry was performed on all cases and association with various clinicopathologic parameters was determined.

**Results:**

Mean age of the patients involved in the study was 48.9 years. Most of the patients presented at stage T2 with a high mean ki67 index i.e. 46.9%. 42.7% of cases had nodal metastasis. Although 84% cases were of invasive ductal carcinoma; however a significant proportion of cases were of metaplastic histology (9.3%). Fifty-one percent (76 cases) of cases showed positive p53 expression while 49% (74 cases) were negative. Higher percentage of p53 expression was found to correlate with higher T stage, high ki67 index and higher nodal stage. On the other hand, strong intensity of p53 expression was positively correlated with higher tumor grade and ki67 index. Seventy-one percent (98 cases) of cases showed positive p16 expression, whereas 24.8% (34 cases) were negative and 3.6% (5 cases) showed focal positive p16 expression. However, no significant association was found between p16 expression and various clinical and pathologic parameters. Similarly, no significant association of either p16 or p53 over-expression was noted with recurrence status of patients.

**Conclusion:**

On the basis of significant association of p53 over-expression with worse prognostic factors in triple negative breast cancer, therefore we suggest that more large scale studies are needed to validate this finding in loco-regional population. Moreover, high expression of p16 in triple negative breast cancer suggests a potential role of this biomarker in triple negative breast cancer pathogenesis which should be investigated with molecular based research in our population.

## Background

Triple negative breast cancers (TNBC) comprise approximately 20% of breast cancers worldwide while a higher frequency of TNBC were noted in south –Asian population [1, 2]. American Society of Clinical Oncology (ASCO)/ College of American Pathologists (CAP) defines TNBC as those breast cancers which shows < 1% estrogen receptor (ER)/ progesterone receptor (PR) expression by immunohistochemistry (IHC) and either 0–1+ Her2neu by IHC or 2+ with negative fluorescent insitu hybridization (FISH) [3–5]. TNBC are typically high grade and associated with worse prognostic and predictive factors and are therefore focus of current clinical research [6, 7]. Moreover TNBC are not a single clinical entity and various subtypes of TNBC have been defined based on molecular studies including basal like subtypes, immunomodulatory, mesenchymal, mesenchymal stem-like, luminal androgen subtypes, claudin low and interferon rich subtypes [8, 9]. Basal like subtype of TNBC is a molecularly defined subtype of TNBC with high expression of basal cytokeratins (CK5/6) and epidermal growth factor receptor (EGFR) and it correlates with IHC expression of CK5/6, [10, 11].

p16 and p53 are proteins which are involved in two major cell cycle control pathways frequently targeted in human tumorigenesis. Virtually all human cancers show dysregulation of either p16 or p53 pathways [12–14]. Prognostic value of p16 and p53 mutations in breast cancer has been shown in various studies [15, 16] however their role as IHC overexpression in TNBC has not been fully understood. Therefore, we aimed to evaluate the association of p16 and p53 overexpression in TNBC with various prognostic parameters like tumor stage, tumor grade, nodal metastasis and lymphovascular invasion.

## Methods

The study included 150 cases of TNBC that had their primary resection at Liaquat National hospital from January 2008 till December 2013 over duration of 6 years. Type of surgeries included wide local excisions and simple mastectomies with sentinel lymph node dissection or wide local excision with axillary dissection and modified radical mastectomies. The approval of the study was taken from institutional research and ethical review committee. At the time of surgery, an informed written consent was taken from each patient. Clinical records of all patients were evaluated and histopathological findings like tumor type, grade and stage were recorded after reviewing H & E slides. Moreover, representative sections of all tumors were re-cut for H & E and IHC staining. ER, PR, Her2neu, Ki67, CK5/6, p16 and p53 IHC were performed on representative sections.

ER, PR, Her2neu and Ki67 IHC were performed using DAKO antibodies as under, with EnVision™ FLEX, high pH DAKO kit according to manufacturer’s protocol.

1. FLEX Monoclonal Rabbit Anti-human Estrogen Receptor alpha, Clone EP1.
2. FLEX Monoclonal Mouse Anti-human Progesterone receptor clone PgR 636
3. Polyclonal Rabbit Anti-human c-erbB-2 oncoprotein
4. FLEX Monoclonal mouse Anti-human Ki67 Antigen clone MIB-1

For ER and PR IHC, nuclear staining in more than 1% cancer cells was taken as positive expression [4]. For, her2neu IHC, staining was scored as per CAP guidelines into 1+ (weak), 2+ (intermediate) and 3+ (strong) expression. Cases with intermediate (2+) expression were subjected to Fluorescent insitu hybridization (FISH) testing and results were reported as amplified or non-amplified as per CAP guidelines [5].

Ki67 IHC was interpreted on the basis of average percentage of positively stained cancer cells. Only nuclear expression was taken as positive. At-least 1000 cancer cells were counted in five different areas of tumor and average percentage of positively stained cancer cells were recorded and then categorized.

CK5/6 IHC was performed by using FLEX Monoclonal Mouse Anti-human Cytokeratin 5/6, clone D5/16 B4 by DAKO envision method according to manufacturers protocol. Moderate to strong cytoplasmic and membranous staining in more than 10% cells was taken as positive expression. Tumors with positive CK5/6 were labeled as basal phenotype and those with negative CK5/6 expression were called as non-basal phenotype.

**Fig. 1:**
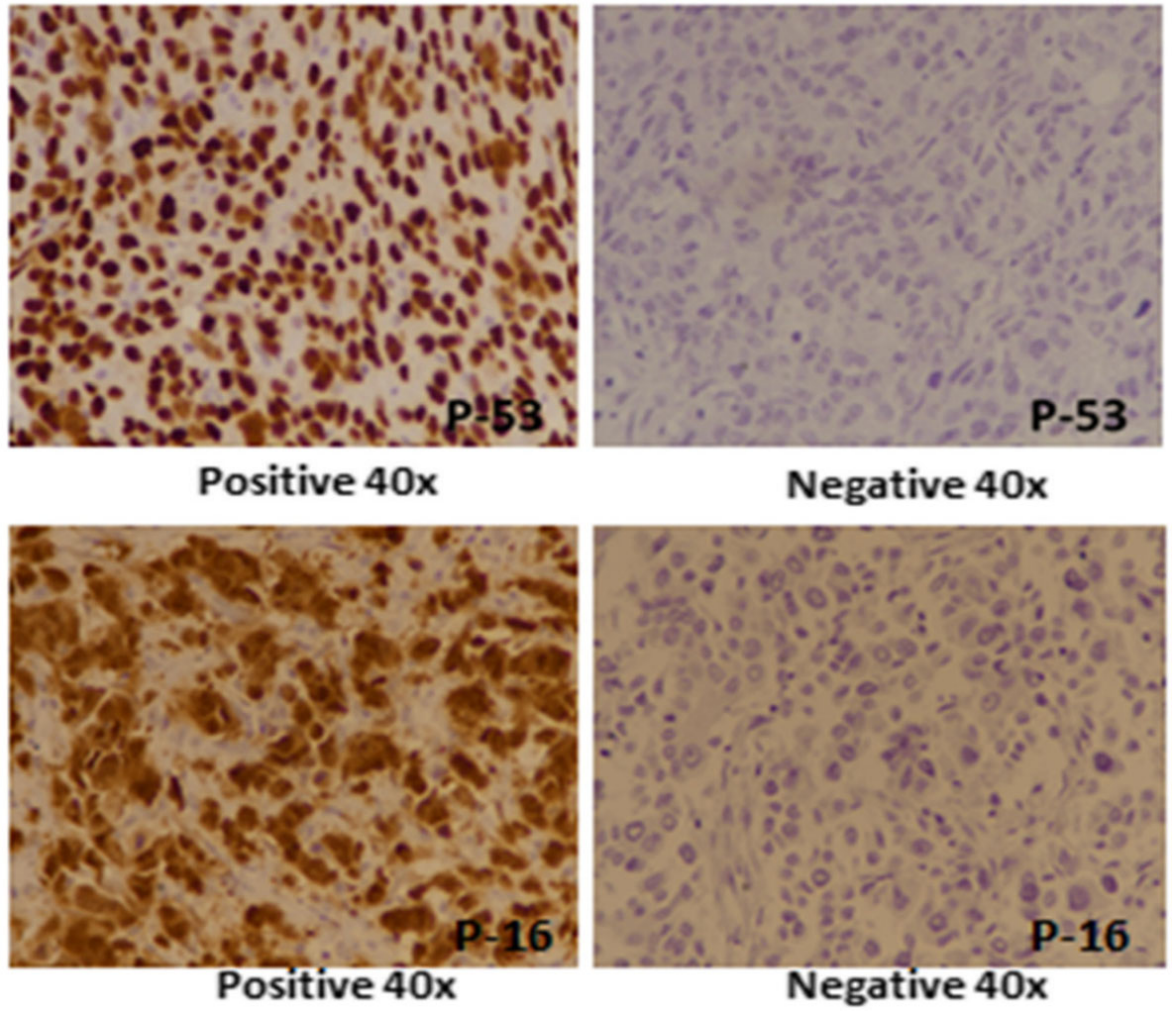
p53 & p16 expression in triple negative breast cancer

p53 IHC was performed using DAKO EnVision method using DAKO anti-human p53 protein, clone DO-7 according to manufacturers protocol. Nuclear staining for p53 was both quantitatively and qualitatively evaluated. Intensity of staining was categorized into no staining (0), weak (1+), intermediate (2+), strong (3+) while percentage of positively stained cells were measured as continuous variable. Intermediate to strong staining in > 10% cancer cells was considered positive while no staining or weak staining in < 10% cancer cells was taken as negative (Fig. 1). Moreover, p53 immunostaining was also categorized according to percentage of staining cells into different groups.

p16 antibody was purchased from Roche Ventana and IHC was performed using antibody CINtec R p16^INK4a^, clone E6H4™ according to manufacturers protocol. Tonsils and carcinoma cervix was taken as positive controls. Both nuclear and cytoplasmic staining was evaluated. Intensity of staining was categorized into no staining (0), weak (1+), intermediate (2+), strong (3+) while percentage of positively stained cells were measured as continuous variable. Intermediate to strong staining in > 10% cancer cells was considered positive while weak to intermediate staining in < 10% cancer cells was taken as focal positive (Fig. 1). Similarly, p16 immunostaining was also categorized according to percentage of staining cells into different groups.

Patient’s clinical records were reviewed to evaluated recurrence and survival status. Time from surgery till death due to disease, local recurrence, distant metastasis or last follow was defined as disease free survival.

Statistical package for social sciences (SPSS 21) was used for data entry and analysis. We calculated mean and standard deviation for quantitative variables while, frequency and percentage were evaluated for qualitative variables. Chi-square was applied to determine association between the variables. Student t test or Mann witney test were applied to compare difference in means among groups where necessary. *P*-value of ≤0.05 was taken as significant. Survival curves were plotted using Kaplan- Meier method and the significance of difference between survival curves were evaluated using log-rank ratio.

A sample size of 150 achieves 79% power to detect an effect size (W) of 0.2994 using a 6 degrees of freedom Chi-Square Test with a significance level (alpha) of 0.05000.

## Results

Mean age of the patients involved in the study was 48.9 years and most common age group was 31–50 years. Most of the patients presented at stage T2 with a high mean ki67 index i.e. 46.9%. 42.7% of cases had nodal metastasis. Although 84% cases were of conventional invasive ductal carcinoma, NST; however a significant proportion of cases were of metaplastic histology (9.3%). Majority cases were of high grade (86.7% grade III). Most tumors show lymphocytic infiltration and necrosis. Most of the tumors lack insitu component (61%) and only 10% cases were of basal phenotype (CK5/6 positive). Local recurrence or late distant metastasis was noted in 17.8% of cases (Table 1).

**Table 1:** Clinicopathologic characteristics of triple negative breast cancer

	n (%)
Age(years)°	48.85 ± 11.49
Age groups	
≤ 30 years	5(3.3)
31–50 years	84(56)
> 50 years	61(40.7)
Tumor size(Unit)°	4.01 ± 1.99
Tumor stage/tumor size	
T1	7(4.7)
T2	116(77.3)
T3/T4	27(18)
Ki67 Index (%)	46.89 ± 23.88
ki67 index groups	
≤ 15%	17(11.3)
16–24%	8(5.3)
25–44%	45(30)
> 44%	80(53.3)
Nodal Status	
Positive	64(42.7)
Negative	86(57.3)
Nodal Stage	
No	88(58.7)
N1	30(20)
N2	13(8.7)
N3	19(12.7)
Histological Subtypes	
IDC	127(84.7)
Papillary	6(4)
Medullary	1(0.7)
Metaplastic	14(9.3)
Mixed	2(1.3)
Tumor Grade	
Grade-I	1(0.7)
Grade-II	19(12.7)
Grade-III	130(86.7)
Lymphocytic infiltration	
Absent	15(10)
Moderate	110(73.3)
Severe	25(16.7)
Lymhovascular Invasion	
Present	36(24)
Absent	114(76)
Dermal Lymphatic invasion	
Present	10(6.7)
Absent	140(93.3)
Type of Surgery	
Modified radical mastectomy	94(62.7)
Simple mastectomy with sentinel lymph node dissection	42(28)
Wide local excision	14(9.3)
Necrosis	
Absent	21(14)
Moderate	90(60)
Severe	39(26)
Fibrosis	
Mild	42(28)
Moderate	88(58.7)
Severe	20(13.3)
Insitu component	
Present	58(38.7)
Absent	92(61.3)
Pagetoid Spread	
Present	2(1.3)
Absent	148(98.7)
Perinodal extension	
Present	30(20)
Absent	120(80)
Triple negative phenotype	
Basal	16(10.7)
Non-basal	134(89.3)
Adjuvant chemotherapy (*n* = 101)	
Yes	98(97)
No	3(3)
Adjuvant radiation(*n* = 101)	
Yes	69(68.3)
No	32(31.7)
Recurrence(*n* = 101)	
Yes	18(17.8)
No	83(82.2)

Mean ± SD

Fifty-one percent (76 cases) of TNBC showed positive p53 expression while 49% (74 cases) were negative. Further categorization on the basis of percentage of p53 expression revealed; 36% (54 cases) showed high p53 expression (> 70%), 12% (18 cases) revealed 51–70% p53 expression, 12% (18 cases) showed 11–50% p53 expression and 40% (60 cases) showed either no p53 expression or weak expression in less than 10% tumor cells. 30.7% (46 cases) showed no p53 expression while 14% (21 cases), 17.3% (26 cases) and 38% (57 cases) revealed weak, intermediate and strong 53 expression respectively. Correlation of percentage of p53 expression with various clinicopathologic variables revealed significant associations (Table 2). High p53 expression was found to correlate with higher T stage, high ki67 index and higher nodal stage. Although not statistically significant, but higher p53 expression was also noted in medullary and metaplastic cancers (*p*-value 0.06). On the other hand, intensity of p53 expression was positively correlated with tumor grade and ki67 index; however, correlation with other parameters was not significant (Table 3).

**Table 2:** Association of percentage of p53 overexpression with various clinical & pathological parameters

	n (%)					*P*-Value
	≤10% (*n* = 60)	11–50% (*n* = 18)	51–70% (*n* = 18)	> 70% (*n* = 54)	Total (*n* = 150)	
Age groups						
≤ 30 years	2(3.3)	0(0)	0(0)	3(5.6)	5(3.3)	0.217
31–50 years	34(56.7)	7(38.9)	8(44.4)	35(64.8)	84(56)	
> 50 years	24(40)	11(61.1)	10(55.6)	16(29.6)	61(40.7)	
Tumor stage/tumor size						
T1(≤2 cm)	3(5)	6(33.3)	3(16.7)	14(25.9)	26(17.3)	0.020
T2(2.1–5.0 cm)	36(60)	6(33.3)	10(55.6)	27(50)	79(52.7)	
T3(> 5.0 cm)	21(35)	6(33.3)	5(27.8)	13(24.1)	45(30)	
ki67 index groups						
≤ 15%	6(10)	6(33.3)	4(22.2)	1(1.9)	17(11.3)	0.000
16–24%	2(3.3)	2(11.1)	3(16.7)	1(1.9)	8(5.3)	
25–44%	19(31.7)	6(33.3)	7(38.9)	13(24.1)	45(30)	
> 44%	33(55)	4(22.2)	4(22.2)	39(72.2)	80(53.3)	
Nodal Status						
Positive	30(50)	5(27.8)	10(55.6)	19(35.2)	64(42.7)	0.144
Negative	30(50)	13(72.2)	8(44.4)	35(64.8)	86(57.3)	
Nodal Stage						
No	32(53.3)	13(72.2)	8(44.4)	35(64.8)	88(58.7)	0.022
N1	15(25)	3(16.7)	2(11.1)	10(18.5)	30(20)	
N2	3(5)	1(5.6)	7(38.9)	2(3.7)	13(8.7)	
N3	10(16.7)	1(5.6)	1(5.6)	7(13)	19(12.7)	
Histological Subtypes						
IDC	51(85)	14(77.8)	12(66.7)	50(92.6)	127(84.7)	0.063
Papillary	1(1.7)	2(11.1)	2(11.1)	1(1.9)	6(4)	
Medullary	0(0)	0(0)	1(5.6)	0(0)	1(0.7)	
metaplastic	7(11.7)	2(11.1)	3(16.7)	2(3.7)	14(9.3)	
Mixed	1(1.7)	0(0)	0(0)	1(1.9)	2(1.3)	
Tumor Grade						
Grade-I	1(1.7)	0(0)	0(0)	0(0)	1(0.7)	0.118
Grade-II	6(10)	6(33.3)	1(5.6)	6(11.1)	19(12.7)	
Grade-III	53(88.3)	12(66.7)	17(94.4)	48(88.9)	130(86.7)	
Lymhovascular Invasion						
Present	13(21.7)	6(33.3)	7(38.9)	10(18.5)	36(24)	0.250
Absent	47(78.3)	12(66.7)	11(61.1)	44(81.5)	114(76)	
Perinodal extension						
Present	12(20)	2(11.1)	6(33.3)	10(18.5)	30(20)	0.436
Absent	48(80)	16(88.9)	12(66.7)	44(81.5)	120(80)	
Triple Negative phenotype						
Basal	6(10)	2(11.1)	2(11.1)	6(11.1)	16(10.7)	1.000
Non Basal	54(90)	16(88.9)	16(88.9)	48(88.9)	134(89.3)	

Chi-Square test applied

*P*-value≤0.05 considered as significant

**Table 3:** Association of intensity of p53 overexpression with various clinical & pathological parameters

	n (%)					*P*-Value
	Weak (*n* = 21)	Intermediate (*n* = 26)	Strong (*n* = 57)	Negative (*n* = 46)	Total (*n* = 150)	
Age groups						
≤ 30 years	0 (0)	0 (0)	3 (5.3)	2 (4.3)	5 (3.3)	0.347
31–50 years	8 (38.1)	14 (53.8)	34(59.6)	28 (60.9)	84 (56)	
> 50 years	13 (61.9)	12 (46.2)	20 (35.1)	16 (34.8)	61 (40.7)	
Tumor stage/tumor size						
T1 (≤2 cm)	6 (28.6)	5 (19.2)	14 (24.6)	1 (2.2)	26 (17.3)	0.023
T2 (2.1–5.0 cm)	9 (42.9)	12 (46.2)	29 (50.9)	29 (63)	79 (52.7)	
T3 (> 5.0 cm)	6 (28.6)	9 (34.6)	14 (24.6)	16 (34.8)	45 (30)	
ki67 index groups						
≤ 15%	5 (23.8)	8 (30.8)	1 (1.8)	3 (6.5)	17 (11.3)	0.006
16–24%	1 (4.8)	2 (7.7)	3 (5.3)	2 (4.3)	8 (5.3)	
25–44%	7 (33.3)	7 (26.9)	16 (28.1)	15 (32.6)	45 (30)	
> 44%	8 (38.1)	9 (34.6)	37 (64.9)	26 (56.5)	80 (53.3)	
Nodal Status						
Positive	9 (42.9)	13 (50)	21 (36.8)	21 (45.7)	64 (42.7)	0.675
Negative	12 (57.1)	13 (50)	36 (63.2)	25 (54.3)	86 (57.3)	
Nodal Stage						
No	12 (57.1)	13 (50)	36 (63.2)	27 (58.7)	88 (58.7)	0.357
N1	5 (23.8)	7 (26.9)	8 (14)	10 (21.7)	30 (20)	
N2	0 (0)	5 (19.2)	5 (8.8)	3 (6.5)	13 (8.7)	
N3	4 (19)	1 (3.8)	8 (14)	6 (13)	19 (12.7)	
Histological Subtypes						
IDC	17 (81)	21 (80.8)	50 (87.7)	39 (84.8)	127 (84.7)	0.620
Papillary	1 (4.8)	1 (3.8)	3 (5.3)	1 (2.2)	6 (4)	
Medullary	0 (0)	1 (3.8)	0 (0)	0 (0)	1 (0.7)	
metaplastic	2 (9.5)	3 (11.5)	3 (5.3)	6 (13)	14 (9.3)	
Mixed	1 (4.8)	0 (0)	1 (1.8)	0 (0)	2 (1.3)	
Tumor Grade						
Grade-I	1 (4.8)	0 (0)	0 (0)	0 (0)	1 (0.7)	0.041
Grade-II	6 (28.6)	4 (15.4)	6 (10.5)	3 (6.5)	19 (12.7)	
Grade-III	14 (66.7)	22 (84.6)	51 (89.6)	43 (93.5)	130 (86.7)	
Lymhovascular Invasion						
Present	7 (33.3)	6 (23.1)	15 (26.3)	8 (17.4)	36 (24)	0.516
Absent	14 (66.7)	20 (76.9)	42 (73.7)	38 (82.6)	114 (76)	
Perinodal extension						
Present	5 (23.8)	3 (11.5)	15 (26.3)	7 (15.2)	30 (20)	0.352
Absent	16 (76.2)	23 (88.5)	42 (73.7)	39 (84.8)	120 (80)	
Triple Negative phenotype						
Basal	3 (14.3)	3 (11.5)	5 (8.8)	5 (10.9)	16 (10.7)	0.913
Non Basal	18 (85.7)	23 (88.5)	52 (91.2)	41 (89.1)	89.3)	

Chi-Square test applied

*P*-value≤0.05 considered as significant

**Fig. 2:**
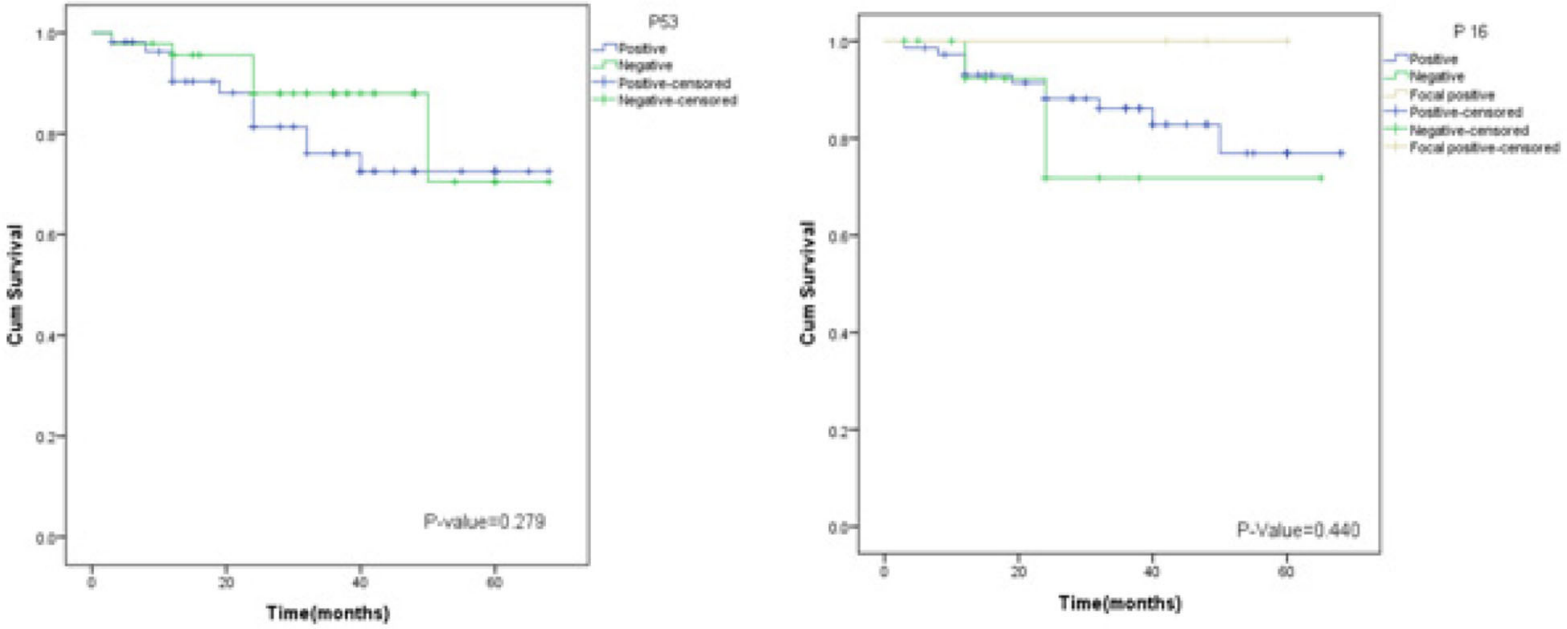
Kalpien-Meier for p53 & p16 overexpression (disease free survival)

Seventy-one percent (98 cases) of TNBC showed positive p16 expression, whereas 24.8% (34 cases) were negative and 3.6% (5 cases) showed focal positive p16 expression. 24.8% (34 cases) revealed no p16 expression while 10.9% (15 cases), 28.5% (39 cases) and 35.8% (49 cases) showed weak, intermediate and strong p16 expression respectively. 28.5% (39 cases) revealed no expression or weak expression in < 10% cancer cells, 15.3% (21 cases) showed 11–50% expression, 13.1% (18 cases) showed 51–70% expression while 43.1% (59 cases) revealed > 70% p16 expression. However, no significant association was found between p16 expression and various clinical and pathologic parameters (Table 3). Similarly, no significant association of either p16 or p53 over-expression was noted with recurrence status of patients (Fig. 2).

## Discussion

In the present study, high expression of p16 was noted in TNBC cases while a moderately high expression of p53 was also notable. Moreover, p53 over-expression significantly correlated with key prognostic factors of breast cancer like T-stage, N-stage, tumor grade and ki67 index.

Breast cancers are quite frequent in Southeast Asia and typically associated with adverse prognostic features [17–20]. Multiple studies investigated the prognostic significance of p53 mutations in breast cancer. Somatic mutations of p53 (TP53) are found in 20–30% of breast cancer [21], while germ-line mutations are relatively rare. Although, the predictive value of TP53 abnormalities is still unclear, somatic TP53 mutations signify worse prognosis independent of tumor size and nodal status [22]. A study involving 1800 patients of breast cancer revealed twice higher risk of death in tumors having TP53 mutations [23]. A similar association of p53 IHC expression with bad prognosis in breast cancer is debatable as cutoff values have not been defined and ASCO panel still don’t advice routine p53 IHC expression testing in breast cancer. However, as mutated p53 protein is not digested quickly inside tumor cells as compared to wild type protein, and therefore accumulates inside tumor cells. Hence, it is reasonable to consider high p53 expression as a surrogate marker of TP53 mutation. Moreover, as various biomarker testing have now been shifted to IHC, therefore with the help of results of various ongoing research, p53 IHC may get incorporated in future ASCO/CAP recommendations. Furthermore, gene expression analysis studies revealed that p53 and other tumor suppressor DNA repair gene mutation and aberrant expression in TNBC may have important clinical implications as they may effect sensitivity to platinum & other chemotherapeutic agents that are directly DNA damaging [24, 25].

Unlike p53, prognostic significance of p16 in TNBC is more controversial; however, high expression of p16 has been noted in various studies [26]. A study involving 60 TNBC cases revealed high ki67 index in p16 positive tumors regardless of p53 expression. As high ki67 index is a well defined prognostic factor in breast cancer [27], therefore they suggested a potential prognostic value of p16 over-expression in TNBC [28]; however, we didn’t find any such association. Basal type phenotype of TNBC is a worse subtype of breast cancer with high expression of CK5/6 (Table 4). Frequency of basal subtype of TNBC in different areas of world is different; we found a low proportion of basal subtype in our study (10%). A study involving 85% of TNBC revealed a high expression of p16 in basal subtype as compared to non-basal phenotype (80% vs. 50.8% respectively) [29]; however, no such association was noted in our study.

**Table 4:** Association of p16 overexpression with various clinical & pathological parameters

	n (%)				*P*-Value
	Positive (*n* = 98)	Negative (*n* = 34)	Focal Positive (n = 5)	Total (*n* = 137)	
Age groups					
≤ 30 years	3(3.1)	0(0)	0(0)	3(2.2)	0.460
31–50 years	59(60.2)	16(47.1)	3(60)	78(56.9)	
> 50 years	36(36.7)	18(52.9)	2(40)	56(40.9)	
Tumor stage/tumor size					
T1(≤2 cm)	16(16.3)	7(20.6)	1(20)	24(17.5)	0.964
T2(2.1–5.0 cm)	51(52)	17(50)	3(60)	71(51.8)	
T3(> 5.0 cm)	31(31.6)	10(29.4)	1(20)	42(30.7)	
ki67 index groups					
≤ 15%	10(10.2)	5(14.7)	2(40)	17(12.4)	0.345
16–24%	5(5.1)	3(8.8)	0(0)	8(5.8)	
25–44%	29(29.6)	11(32.4)	2(40)	42(30.7)	
> 44%	54(55.1)	15(44.1)	1(20)	70(51.1)	
Nodal Status					
Positive	42(42.9)	15(44.1)	2(40)	59(43.1)	1.000
Negative	56(57.1)	19(55.9)	3(60)	78(56.9)	
Nodal Stage					
No	58(59.2)	19(55.9)	3(60)	80(58.4)	0.907
N1	17(17.3)	8(23.5)	2(40)	27(19.7)	
N2	9(9.2)	3(8.8)	0(0)	12(8.8)	
N3	14(14.3)	4(11.8)	0(0)	18(13.1)	
Histological Subtypes					
IDC	83(84.7)	28(82.4)	5(100)	116(84.7)	0.633
Papillary	5(5.1)	0(0)	0(0)	5(3.6)	
Medullary	1(1)	0(0)	0(0)	1(0.7)	
metaplastic	8(8.2)	5(14.7)	0(0)	13(9.5)	
Mixed	1(1)	1(2.9)	0(0)	2(1.5)	
Tumor Grade					
Grade-I	0(0)	1(2.9)	0(0)	1(0.7)	0.165
Grade-II	11(11.2)	7(20.6)	0(0)	18(13.1)	
Grade-III	87(88.8)	26(76.5)	5(100)	118(86.1)	
Lymhovascular Invasion					
Present	25(25.5)	6(17.6)	1(20)	32(23.4)	0.788
Absent	73(74.5)	28(82.4)	4(80)	105(76.6)	
Perinodal extension					
Present	19(19.4)	9(26.5)	0(0)	28(20.4)	0.425
Absent	79(80.6)	25(73.5)	5(100)	109(79.6)	
Triple Negative phenotype					
Basal	10(10.2)	3(8.8)	1(20)	14(10.2)	0.532
Non Basal	88(89.8)	31(91.2)	4(80)	123(89.8)	

Chi-Square test applied

*P*-Value≤0.05, considerd as significant

One of the limitations of our study was that molecular testing of p16 & p53 was not performed, therefore we suggest molecular testing of p16 & p53 in TNBC of our population to establish mutation status and its correlation with IHC over-expression of these biomarkers. Moreover, we didn’t find any significant correlation of recurrence status of TNBC with p53 &p16 over-expression; however it can’t be concluded that there is no correlation of p53 expression with recurrence status, as other important factors determining recurrence like margin status of tumors was not taken into account.

## Conclusion

On the basis of significant association of p53 IHC over-expression with worse prognostic factors in TNBC, therefore we suggest that more large scale studies are needed to validate this finding in loco-regional population. Moreover, high expression of p16 in TNBC suggests a potential role of this biomarker in TNBC pathogenesis which should be investigated with molecular based research in our population.
